# **Structural Defects Associated with Craniectomy Induce Neuroinflammation and Blood**–**Brain Barrier Permeability**

**DOI:** 10.1177/08977151251362176

**Published:** 2025-07-26

**Authors:** Aria W. Tarudji, Brandon Z. McDonald, Evan Curtis, Connor Gee, Forrest M. Kievit

**Affiliations:** Department of Biological Systems Engineering, University of Nebraska–Lincoln, Lincoln, Nebraska, USA.

**Keywords:** controlled cortical impact, craniotomy, naïve, sham

## Abstract

Heterogeneity associated with traumatic brain injury (TBI) outcomes necessitates validated controls to differentiate pathophysiological events from experimental methodology. While craniectomies are commonly used in TBI research, inadvertent dura disruption can result in structural deficits, impacting cellular function and neurobehavioral outcomes. Thus, there is a critical need to evaluate the effect of craniectomy on neurological outcomes to develop robust experimental controls and improve pre-clinical TBI research. In this study, craniectomy mice undergoing surgical and anesthetic intervention were assessed against naïve mice for neurological deficits and pathophysiological dysfunction. T2-weighted magnetic resonance imaging confirmed that no lesions or cavities were observed postcraniectomy. However, the cranial defect induced midline shifting over time, which might contribute to poorer behavioral outcomes in the novel object recognition assessment. Immunohistochemical analysis demonstrated an increase in GFAP and Iba1, indicating craniectomy elicited an inflammatory response. Indeed, neuroinflammation led to an increase in neuronal cell death, as measured by increases in α-II-spectrin breakdown products. However, craniectomy mice also presented with decreases in LC3BII and SQSTM1 expression, indicating an inhibition of autophagy. Last, craniectomy contributed to the altered expression of several tight junction proteins, including occludin and claudin-1/5, suggesting the blood–brain barrier was perturbed. Overall, the deficits associated with craniectomy preclude its use as an adequate sole control for TBI research, as craniectomy limits translational insights into the neurological changes observed in TBI. Additionally, these results support the need for the use of closed-head injury models where uninjured control mice do not show significant confounding minor injury patterns.

## Introduction

Complex outcomes associated with traumatic brain injury (TBI) require robust experimental controls to differentiate between injury sequelae and surgical intervention. Craniectomies serve as a common surgical approach in TBI research, facilitating access to the impact site.^1–3^ However, producing a cranial window is challenging since dura disruption leads to structural and biochemical consequences, contributing to poor behavioral outcomes.^4,5^ The initial cranial defect may inadvertently introduce confounding variables that complicate data interpretation to inform clinical translation. Thus, the question lies in whether the effect of craniectomy is reproducible and serves as an adequate experimental control for TBI research.

The neurological effects and complications of craniectomy have been examined clinically^6–13^ and in pre-clinical animal models.^4,5,14^ Clinical assessments concluded that craniectomy, as an intervention for improving mortality following trauma, could exacerbate morbidity.^7–9^ Additionally, pre-clinical studies demonstrated that a cranial defect contributes to neurological deficits. T1- and T2-weighted magnetic resonance imaging (MRI) confirmed that surgical manipulation of the skull induced detectable changes in brain structure, regardless of craniectomy method.^4,5^ Ultimately, these structural deficits induced an inflammatory response and resulted in poor neurobehavioral outcomes, including seizures. These effects confound efforts to model disease and assess therapeutic strategies. Thus, elucidating the impact of surgical intervention, without dural damage, on experimental outcomes is critical for developing suitable models and improving the translational relevance of pre-clinical TBI research.^14^

In this study, craniectomy mice subjected to anesthesia and craniectomy were evaluated against naïve mice for neurological deficits and pathophysiological dysfunction. Craniectomy mice were examined for gross neuropathological features and structural defects utilizing T2-weighted MRI. Behavioral outcomes were assessed via novel object recognition (NOR). Immunohistochemistry (IHC) was used to monitor changes in glial fibrillary acidic protein (GFAP) and ionized calcium-binding adapter molecule 1 (Iba1). Male and female craniectomy mice were evaluated using immunoblotting (IB) for changes in neuronal function, including phosphorylated tau and α-II-spectrin breakdown products. Time course changes in autophagy proteins, LC3BII and sequestosome-1 (SQSTM1), were evaluated to examine protein turnover. Last, a panel of tight junction proteins, including occludin and claudin-1/5, was assessed using IB for changes in blood–brain barrier (BBB) integrity.

## Materials and Methods

### Antibodies

Primary and secondary antibodies used for IHC and IB are provided in Tables 1 and 2.

**Table 1.** Primary and Secondary Antibodies Utilized for Immunohistochemistry

	Host species	Company, catalog no.
Primary antibody		
GFAP	Goat	Abcam, ab53554
Iba1	Rabbit	Wako, 019-19741
NeuN	Guinea Pig	Sigma Aldrich, ABN90P
Secondary antibody		
Anti-rabbit	Donkey	Abcam, ab150074
Anti-goat	Donkey	Abcam, ab150129
Anti-guinea pig	Donkey	Jackson, 706-605-148

**Table 2.** Primary and Secondary Antibodies Utilized for Immunoblotting

	Host species	Company, catalog no.
Primary antibody		
β-Actin (loading control)	Mouse	Sigma Aldrich, A2228
GFAP	Rabbit	Abcam, ab7260
pTau	Rabbit	Abcam, ab81268
α-II Spectrin	Mouse	Sigma Aldrich, MAB1622
SQSTM1	Mouse	Abcam, ab56416
LC3B	Rabbit	Abcam, ab192890
Bax	Rabbit	Cell Signaling Technology, 2772S
IgG	Goat	BioRad, 1705047
Occludin	Rabbit	Thermo Fisher, 71-1500
Claudin1	Rabbit	Abcam, ab15098
Claudin5	Mouse	Thermo Fisher, 35-2500
Secondary antibody		
Anti-rabbit	Goat	BioRad, 1705046
Anti-mouse	Goat	BioRad, 1705047

### Craniectomy procedure

**FIG. 1.**
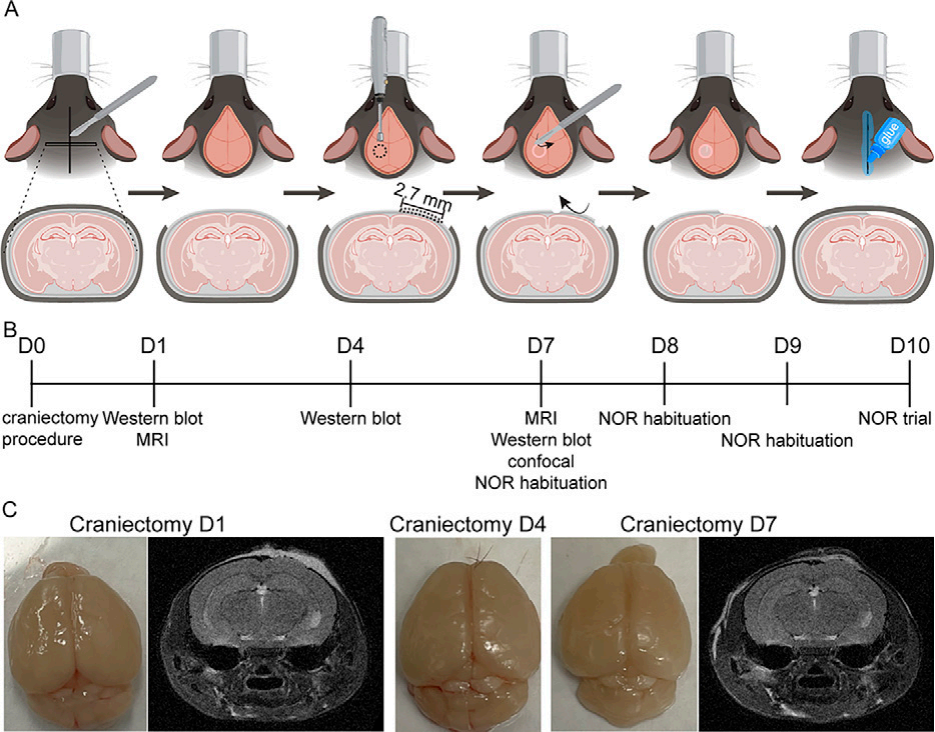
Overview of the craniectomy procedure and experimental design. **(A)** An overview of the craniectomy procedure, including hair removal, craniectomy, and wound annealing via surgical adhesive. Mice with evidence of dura disruption during the surgery were removed from the study. **(B)** An overview of the experimental design and sample collection. **(C)** Representative images of the excised brains and coronal sections of T2-weighted MRI images showed no noticeable damage on days 1, 4, and 7 postcraniectomy. MRI, magnetic resonance imaging; NOR, novel object recognition.

All experimental procedures were conducted in accordance with the University of Nebraska–Lincoln Animal Care and Use Committee (UNL IACUC #2300). Eight-week-old male and female C57BL/6J mice (Jackson Laboratory, Bar Harbor, ME) underwent craniectomy surgeries as described previously, without head impact.^15–17^ Briefly, mice were anesthetized and positioned in a stereotaxic frame (Model 963, David Kopf Instruments), with 2.0% isoflurane via inhalation. Following hair removal, the surgical site was prepared with iodine, followed by a 1 cm midline scalp incision and a 2.7 mm craniectomy, centered at 2 mm posterior to bregma and 2 mm lateral to the midline. The incision was closed using surgical adhesive. Figure 1A provides a schematic representation of the craniectomy procedure.

### In vivo MRI

Measurements for lesion and cavity sizes were modified from previous studies.^18,19^ Mice were scanned at 1 and 7 days postcraniectomy on a 9.4T MRI system (Varian, Palo Alto, CA) with a 4-cm Millipede RF imaging probe and triple-axis gradients (100 G/cm max, 1000 mT/m). During imaging, mice were anesthetized with 1.0–1.5% isoflurane. T2-weighted images were acquired with a fast spin-echo multislice sequence (FSEMS) (Table 3).^18^ The images were processed on VnmrJ 3.0c software (Agilent, Santa Clara, CA). Lesion and cavity regions were drawn by a blinded experimentalist using ITK-SNAP software (ver 3.6) around a hyperintense signal and hypointense signal relative to the surrounding tissue, respectively.^19^ Midline shift was quantified by a blinded experimentalist using ImageJ by measuring the distance between the center of the third ventricle and the center line of the skull.

**Table 3.** Parameters for Fast Spin-Echo Multislice Sequence for T2-Weighted MRI

Fast spin-echo multislice sequence (FSEMS) parameters	
Repetition time (TR)	5500 ms
Echo spacing	12.5 ms
Echo train length/no. of segments (ETL/Seg)	64/4
K-zero	4
Effective echo time	50.02 ms
Averages	2
Matrix size	256 × 256
Field of view	20 mm × 20 mm
Slice thickness	0.5 mm
No. of slices	15
Effective in-plane resolution	78 μm × 78 μm
Scan time	11 min 58 s

### Novel object recognition

All behavioral testing was conducted on female mice 7 days postcraniectomy. The mice were habituated in an open field chamber without objects for 3 days, 10 min each, to prevent anxiety-like behaviors from exposure to the environment. On the fourth day (10 days postcraniectomy), two identical objects were placed in the northeast and southeast quadrants of the open field, 2 cm from the respective corners. After a 4-hour intertrial interval, the object in the northeast quadrant was replaced with a novel object. The mice explored identical and novel objects for 10 min. The time spent exploring each object was measured and used to determine the discrimination index (DI) (Equation 1 in Supplementary Data).

### Immunostaining

Methodology describing cryosection preparation can be found in the supplemental methods (Supplementary Fig. S1). Slides underwent three phosphate-buffered saline (PBS) rinses and 1-h blocking RT (PBS containing 3% normal donkey serum, 0.3% Triton X-100, 0.1% sodium azide). Primary antibody incubation was performed at 4°C for 24 h, followed by blocking buffer washes (3 × 5 min). Secondary antibody incubation proceeded for 1.5 h at RT. After blocking buffer washes (3 × 5 min), sections were counterstained with 4′,6-diamidino-2-phenylindole (DAPI) (5 min), washed twice with PBS, and once with ddH_2_O. Sections were mounted using ProLong™ Gold antifade and stored overnight at RT, then at 4°C until imaging. Images were acquired using a confocal microscope (LSM800, Zeiss) with 5x, 10x, and 20x magnification. GFAP+ astrocytes and Iba1+ microglia were counted manually in ImageJ and divided by the total image area (mm^2^) for region density.

### Western blot

Methodology describing lysate preparation can be found in the supplemental methods (Supplementary Fig. S2). Proteins were separated by electrophoresis (120 V, 80 min) and transferred to polyvinylidene fluoride (PVDF) membranes using Trans-Blot® Turbo™. After tris-buffered saline (TBS) washing (2 × 5 min), membranes were blocked [(5% Blot-Quick Blocker™ or bovine serum albumin (BSA) in TBS + 1% Tween® 20 (TBST)] for 1 h at RT, incubated with primary antibodies at 4°C overnight, washed (TBST, 3 × 5 min), and incubated with secondary antibodies (1 h). Following TBST washes (3 × 10 min), membranes were developed with enhanced chemiluminescence (ECL) reagent and imaged.

### Statistical analysis

All statistical analyses and graphs were performed and generated using GraphPad Prism (version 9.3.1). Power analysis was used to determine the required number of replicates based on our previous *in vivo* studies.^15–17^ The effect size between naïve and craniectomy was evaluated using the Student’s *t*-test, while postcraniectomy time course analysis was analyzed using one-way or two-way analysis of variance (ANOVA) with Sidak’s multiple comparisons test or Tukey’s Honest Significant Difference test for *post hoc* analysis.

## Results

### Craniectomy creates structural deficits without gross neuropathological features

**FIG. 2.**
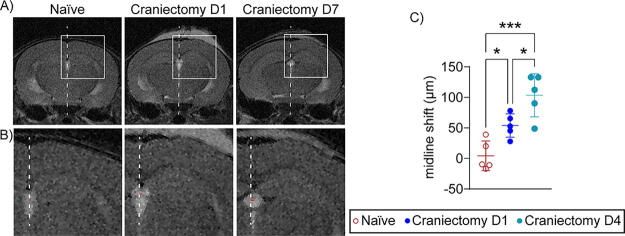
Craniectomy induces midline shifting in mouse brains. **(A)** Representative T2 images were taken from naïve and craniectomy male mice (*n* = 5) at 1 and 7 days postcraniectomy. The white dashed line represents the true midline, and the boxed area represents an inlay for magnified images. **(B)** Magnified images to highlight the midline shift between treatment groups. The white dashed line represents the true midline, and the red line represents the shift in the midline. **(C)** Quantification for the distance in midline shift for naïve and days 1 and 7 postcraniectomy (*n* = 6). We observed a significant midline shift on day 7 postcraniectomy compared to day 1 craniectomy and naïve. Data are shown as mean ± SD with * and *** representing *p* < 0.05 and *p* < 0.001, respectively, as determined by one-way ANOVA and Tukey’s *post hoc* test. SD, standard deviation.

An experimental timeline for assessing neurological differences between naïve and craniectomy mice is provided below (Fig. 1B). Owing to the risk of disrupting the dura (Supplementary Figs. S1, Figs. S5), mice were perfused at 1, 4, and 7 days postcraniectomy, and gross neuropathology was assessed. No lesions or hemorrhaging were observed for each mouse (Fig. 1C, Supplementary Fig. S1). However, we anticipated that craniectomy mice may experience mild structural differences as a result of ineffective craniectomy.^4,5,10^ Five male craniectomy mice were evaluated using MRI, and T2-weighted images validated that neither lesions nor cavities were present on day 1 or 7 postcraniectomy (Supplementary Fig. S2). However, images revealed that a portion of brain tissue had protruded through the cranial window, which might be caused by positive intracranial pressure with intact dura,^20^ inducing a significant shifting of the midline over time (Figs. 1C, 2). Indeed, cranial protrusion has been reported clinically as a complication of decompressive craniectomy.^10–13^

### Craniectomy affects recognition memory in NOR

**FIG. 3.**
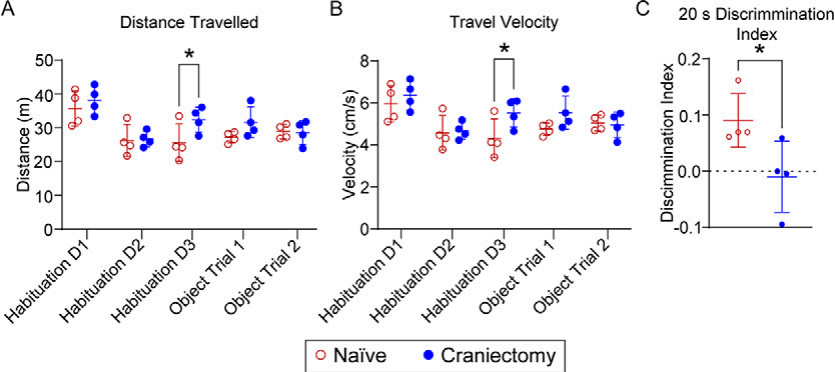
Craniectomy affects recognition memory in NOR assessment. Female mice (*n* = 4) were habituated for 3 days (days 7–9 postcraniectomy), and NOR was assessed on day 10 postcraniectomy. We observed a significant increase in distance travelled **(A)** and travel velocity **(B)** on habituation trial 3. **(C)** Additionally, craniectomy mice demonstrated a significant deficit in performance in discriminating between novel and familiar objects when compared to naïve mice (*p* < 0.05). Data are represented as mean ± SD. Two-way ANOVA analysis with Sidak’s multiple comparison test was performed for distance travelled and travel velocity, while Student *t-*test with Mann–Whitney *post hoc* analysis was performed for assessing the discrimination index. NOR, novel object recognition.

Structural abnormalities in craniectomy mice may cause behavioral deficits. The cognitive behavior of female craniectomy mice was evaluated using NOR.^21,22^ Craniectomy mice demonstrated a deficit in performance to discriminate between novel and familiar objects, suggesting a functional deficit in recognition memory. We observed a significant difference in both travel distance and velocity on habituation trial 3, which might suggest an increase in anxiety-like behavior postcraniectomy (Fig. 3). We appreciate that the number of animals is extremely underpowered for a behavioral study, which might be impacted by cage effect and type I error. To ensure these factors did not confound the DIs, we examined total exploration time. We observed no significant differences between naïve and craniectomy, suggesting intact motor coordination, no anxiety-like effect, and minimal cage effect on exploration (Supplementary Fig. S3).

### Craniectomy exacerbates glial activity

**FIG. 4.**
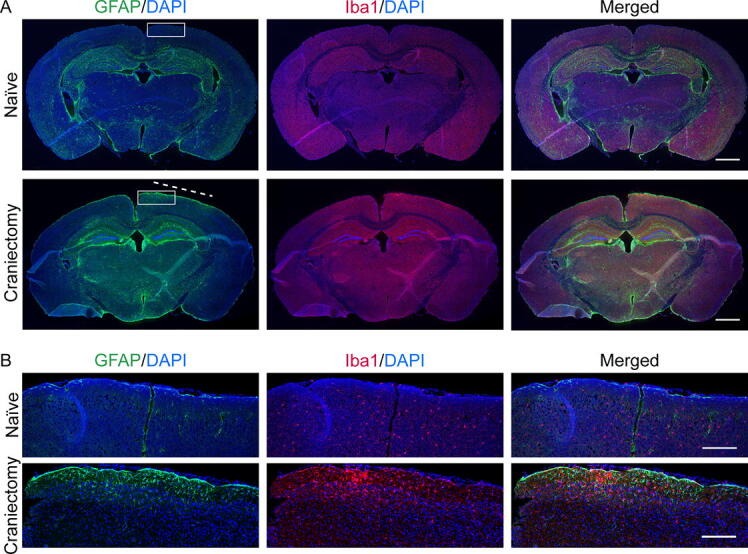
Craniectomy procedure induces reactive astrocytes and activated microglia. **(A)** Fluorescence microscopy of the whole brain of naïve and day 7 craniectomy mice with GFAP (astrocyte), Iba1 (microglia), and DAPI (nuclei) staining taken with a 5x magnification lens. The boxed area represents a magnified area of the cortex. The dashed line is a representative 2.5 mm scale for the diameter of the 2.7 mm trephine. Scale bars correspond to 1000 μm. **(B)** Fluorescence microscopy of the cortex of naïve and craniectomy mice with GFAP, Iba1, and DAPI staining taken with a 10x magnification lens. Scale bars correspond to 200 μm. Color code: Green—GFAP, red—Iba1, and blue—DAPI.

**FIG. 5.**
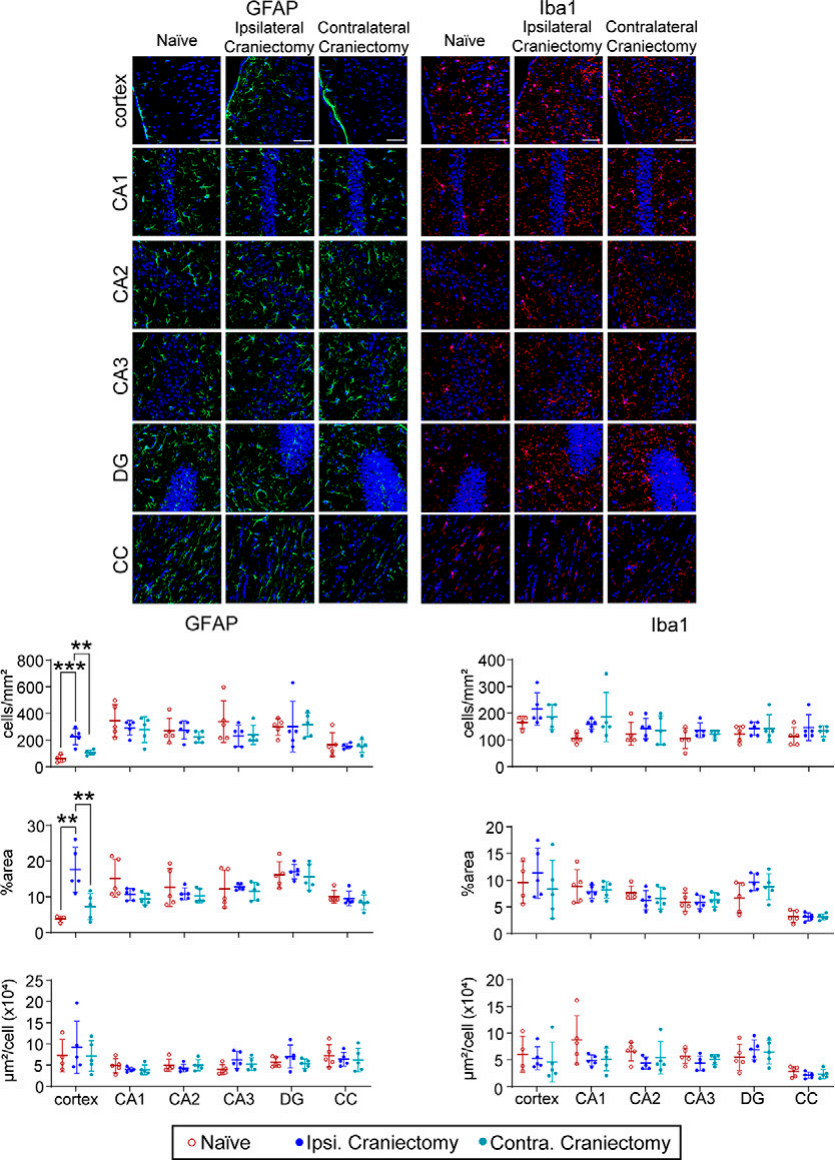
Craniectomy procedure induces reactive astrocytes and trending activation of microglia in the cortex. Representative confocal images of GFAP and Iba1 in the cortex, hippocampus, and corpus callosum of naïve and day 7 craniectomy male mice. In the ipsilateral cortex, we observed a significant increase in cell density and size of astrocytes and a trending increase in cell density of microglia. Data are shown as mean ± SD with ** and *** representing *p* < 0.01 and *p* < 0.001, respectively, as determined by two-way ANOVA and Tukey’s *post hoc* test. Magnification lens: 20x. Scale bar: 50 μm. Color code: Green—GFAP, red—Iba1, and blue—DAPI. SD, standard deviation.

The skull is essential in regulating the brain’s immune response through physical and biological mechanisms.^23^ Several studies demonstrated the anti-inflammatory effect of myeloid cells derived from skull bone marrow, suggesting that cranial defects have the potential to elicit immune responses.^24,25^ Thus, we assessed astrocyte and microglia activity using IHC at 7 days postcraniectomy, corresponding with the greatest midline shift. The quantification revealed a significant increase in astrocyte density in the ipsilateral cortex at 7 days postcraniectomy (Figs. 4, 5, Supplementary Fig. S4). Additionally, we observed a trending increase in Iba1 in the ipsilateral cortex and ipsilateral dentate gyrus. We noted a possible increase in immunoreactivity within the thalamus and hypothalamus of craniectomy mice, potentially related to midline shift. This observation, however, was not explored in greater depth due to the established rarity of thalamic and hypothalamic involvement in mouse models of TBI.

### Craniectomy affects glial and neuronal function

**FIG. 6.**
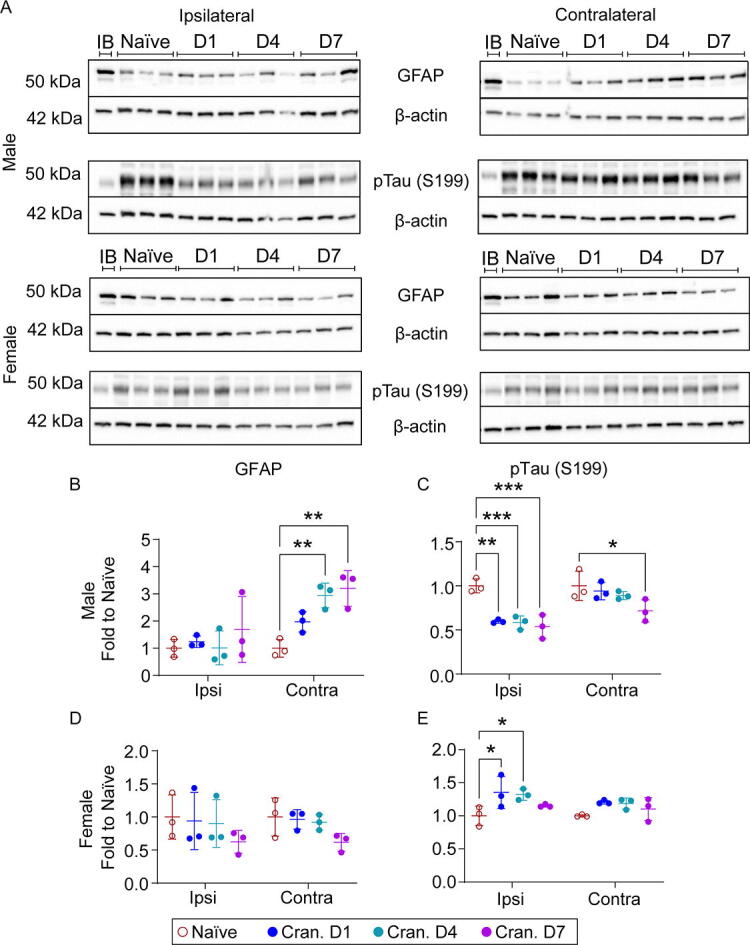
Craniectomy procedure impacts GFAP and Tau phosphorylation. **(A)** Representative Western blot of GFAP and Tau phosphorylation (S199) in the cortex of naïve and days 1, 4, and 7 craniectomy male and female mice. In craniectomy male mice **(B)**, we observed a significant increase in GFAP in the contralateral cortex at 4 and 7 days, but not in female mice **(C)**. Additionally, we observed a significant decrease in phosphorylated tau (S199) in both the ipsilateral and contralateral cortex in male mice **(D)**, while in female craniectomy mice **(E)**, we observed a significant increase at 1 and 4 days. Data are shown as mean ± SD with *, **, and *** representing *p* < 0.05, *p* < 0.01, and *p* < 0.001, respectively, as determined by two-way ANOVA and Tukey’s *post hoc* test. IB, interblot control; SD, standard deviation.

IHC confirmed increased GFAP in the ipsilateral cortex at 7 days postcraniectomy. Thus, we examined time course changes in GFAP via IB in the cortices from both male and female mice. We observed a significant increase in GFAP in the contralateral cortex of male mice at 4 and 7 days postcraniectomy (Fig. 6). No significant differences were observed in either region for female mice, with a trending decrease in levels over time. Time course changes in phosphorylated tau (pTau) revealed a significant increase in pTau (S199) in female mice in the ipsilateral cortex at 1 and 4 days postcraniectomy, suggesting craniectomy impacts neuronal function. Interestingly, we observed a significant reduction in pTau in the ipsilateral cortex for male mice at each time point.

**FIG. 7.**
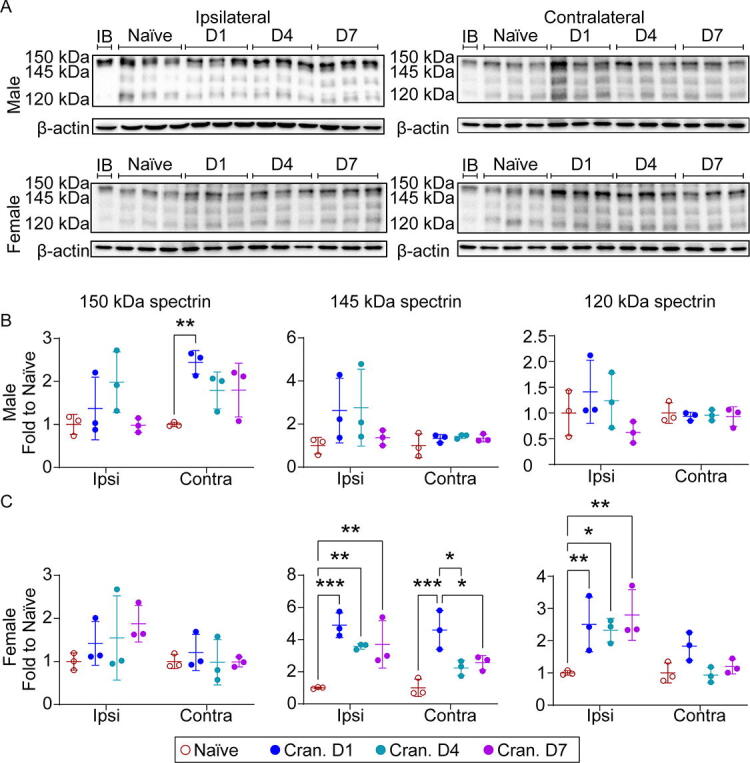
Craniectomy procedure induces cell death in the cortex. **(A)** Representative Western blot of α-II-spectrin breakdown products (SBDPs) in the cortex of naïve and days 1, 4, and 7 craniectomy male and female mice. **(B)** In male mice, we observed a significant increase in 150 kDa SBDPs in the contralateral cortex on day 1. **(C)** In female mice, we observed a significant increase in 145 and 120 kDa SBDPs in the ipsilateral cortex. Data are shown as mean ± SD with *, **, and *** representing *p* < 0.05, *p* < 0.01, and *p* < 0.001, respectively, as determined by two-way ANOVA and Tukey’s *post hoc* test. IB, interblot control; SD, standard deviation.

Differences in tau phosphorylation indicated that craniectomy mice elicit a dysfunctional neuronal phenotype, which may result from cell death. We investigated time course changes in α-II-spectrin breakdown products (SBDPs) to monitor levels of neuronal cell death via necrosis (145 kDa) or apoptosis (120 kDa) for both male and female mice. In male craniectomy mice, we observed a significant increase in total cell death (150 kDa) in the contralateral cortex at day 1, with a trending increase in both necrosis and apoptosis (Fig. 7). Interestingly, female craniectomy mice exhibited a peak in necrosis in both the ipsilateral and contralateral cortex, with a significant increase over time in apoptosis in the ipsilateral region.

### Craniectomy inhibits autophagy

**FIG. 8.**
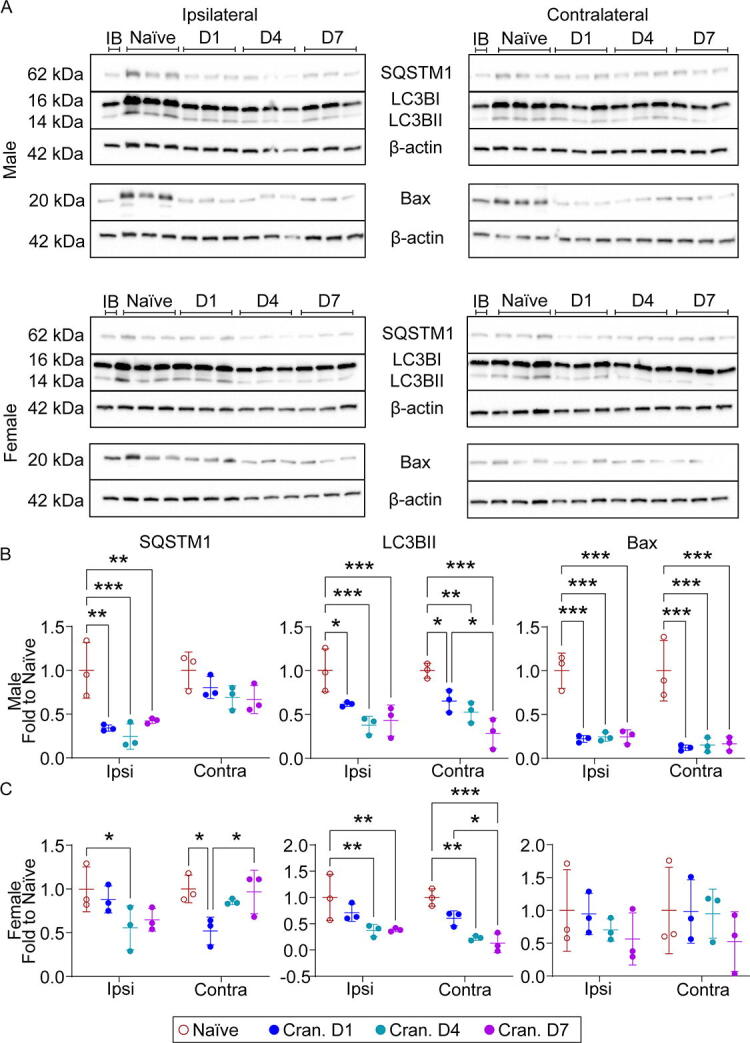
Craniectomy procedure inhibits autophagy. **(A)** Representative Western blot of SQSTM1, LC3B, and Bax in the cortex of naïve and day 1, 4, and 7 craniectomy male and female mice. **(B)** In male mice, we observed a significant decrease in SQSTM1, LC3BII, and Bax in the ipsilateral and contralateral cortex at 1, 4, and 7 days postcraniectomy. **(C)** In female mice, we observed a significant reduction in SQSTM1 and LC3BII at 4 days postcraniectomy. Data are shown as mean ± SD with *, **, and *** representing *p* < 0.05, *p* < 0.01, and *p* < 0.001, respectively, as determined by two-way ANOVA and Tukey’s *post hoc* test. IB, interblot control; SD, standard deviation.

Autophagy contributes to TBI pathophysiology,^17,26,27^ owing to its role in regulating the immune response.^28–30^ Thus, we examined the effect of craniectomy on autophagic flux and apoptosis. We observed a significant decrease in both SQSTM1 and LC3BII in the ipsilateral cortex from male mice at each time point and a significant decrease at day 4 in female mice, suggesting craniectomy inhibits autophagy (Fig. 8). Interestingly, we observed a significant and trending decrease in the apoptotic regulator, Bax, in brain regions of male and female mice, respectively. Overall, these results suggested that the neuroinflammatory response may be associated with a reduction in autophagy postcraniectomy.

### Craniectomy perturbs the BBB

**FIG. 9.**
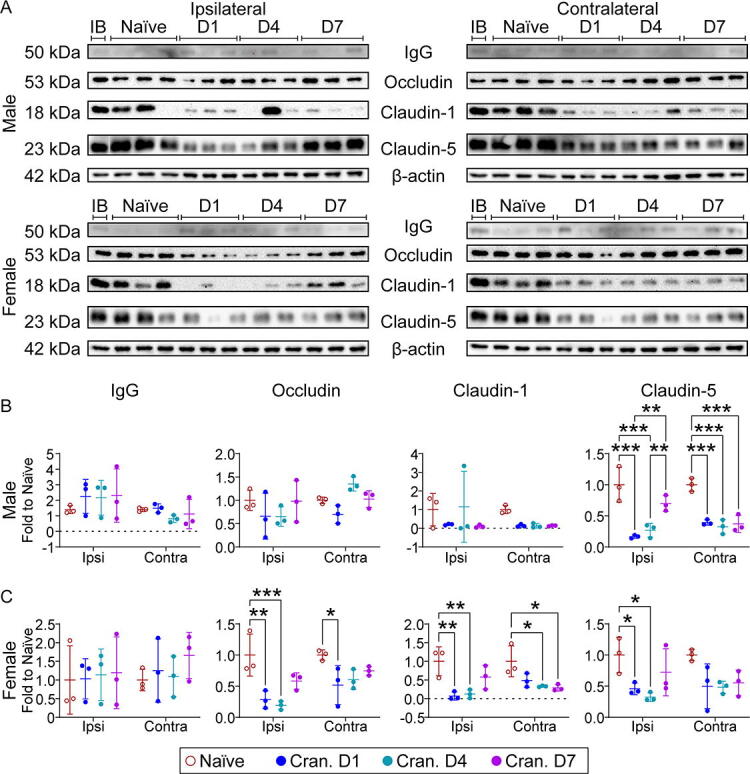
Craniectomy procedure induces blood–brain barrier (BBB) disruption. **(A)** Representative Western blot of IgG, occludin, claudin1, claudin5, and β-actin in the cortex of naïve and day 1, 4, and 7 craniectomy male and female mice. **(B)** In male mice, we observed a significant decrease in claudin5 expression following the craniectomy procedure. **(C)** In female mice, we observed a significant decrease in occludin, claudin1, and claudin5 following the craniectomy procedure. Data are shown as mean ± SD with *, **, and *** representing *p* < 0.05, *p* < 0.01, and *p* < 0.001, respectively, as determined by two-way ANOVA and Tukey’s *post hoc* test. IB, interblot control.

Given the significance of BBB disruption in drug delivery and active targeting for neurological diseases, we assessed the effect of craniectomy on BBB disruption. We examined time course changes in IgG and the tight junction proteins—occludin, claudin-1, and claudin-5 (Fig. 9). We observed a trending increase in IgG in the ipsilateral cortex for both male and female craniectomy mice. In male craniectomy mice, claudin-5 was significantly downregulated at each time point in both the ipsilateral and contralateral cortices. Female craniectomy mice had a significant reduction in occludin and claudin-1 for both regions, with a significant decrease in claudin-5 in the ipsilateral cortex. Together, these data suggested that the craniectomy procedure disrupts the BBB.

## Discussion

Experimental controls are critical for validating disease models and assessing therapeutic intervention. For TBI, several animal models require a craniectomy,^1–3^ which risks disrupting the dura and confounding interpretations of injury sequelae and behavioral function, as found in Supplementary Figure S5 and previous publications.^4,5^ Here, we demonstrated a craniectomy procedure without dura disruption, as supported by the absence of gross neuropathological features (Fig. 1C, Supplementary Fig. S1), lesion and cavity (Supplementary Fig. S2), changes in urinary 8-isoprostane levels (Supplementary Fig. S6), and impact on body weight (Supplementary Fig. S7). However, we observed neurological deficits and pathophysiological dysfunction, including midline shift and neuroinflammation in craniectomy male mice (Fig. 2), as well as DIs deficit in craniectomy female mice (Fig. 3), suggesting a functional deficit in recognition memory. We also observed cellular homeostasis impairment and BBB permeability in both sexes. Although the body size difference between male and female mice is noted, the lack of significant brain and skull size differences (2.5%) suggests that skull bone structure is unlikely to explain the sex-specific findings in our experiment.^31,32^ Thus, these results demonstrated that the surgical procedure alone affects behavior, which confounded impact-mediated outcomes. Owing to their role in memory consolidation, this study focused on the cortex and hippocampus.

Both craniectomy and anesthesia influence the immune system.^13,33–35^ IHC results confirmed an increase in reactive astrocytes and a trending increase in activated microglia in the ipsilateral cortex at 7 days postcraniectomy (Figs. 4, 5), corresponding with the greatest increase in midline shifting (Fig. 2). Although only male mice were used for IHC, time course changes in GFAP expression were conducted with IB in both sexes (Fig. 6). Male craniectomy mice had a significant increase in GFAP at 4 and 7 days postcraniectomy in the contralateral cortex but not in the ipsilateral cortex. These findings from IHC and IB aligned, demonstrating a two- to threefold increase of GFAP in the contralateral cortex. In the ipsilateral cortex, regional IHC analysis, focused on the craniectomy site, revealed a significant increase in GFAP, but it was masked when the whole ipsilateral cortex was analyzed via IB. In female craniectomy mice, a trending decrease in GFAP over time may result from the effect of anesthesia on astrocyte morphology and function.^36,37^ These differences demonstrate an effect with respect to sex on neuroinflammation postcraniectomy, suggesting altered neuronal function.

Time course changes in pTau S199 revealed a significant increase in female mice in the ipsilateral cortex at 1 and 4 days postcraniectomy (Fig. 6), which correlates with previous literature investigating the effects of anesthetic agents on tau phosphorylation.^38,39^ Interestingly, we observed a significant reduction in pTau (S199) in the ipsilateral cortex for male mice at each time point. Anesthesia can inhibit excitatory neurons, which represent the majority of neuronal activity in the mouse cortex,^40^ and reduce markers of synaptic activity, including synaptophysin and postsynaptic density protein-95,^41^ which could decrease tau phosphorylation. Differences in tau phosphorylation between sexes may result from the effect of 17-β estradiol on mitogen-activated protein kinase (MAPK) signaling.^42,43^ Indeed, JNK activation is higher in female tauopathy mice.^44^ Overall, these changes in tau phosphorylation demonstrated that craniectomy compromises neuronal homeostasis, which may result in cell death.

Time course changes in SBDPs revealed a significant increase in total cell death in the contralateral cortex of male mice at day 1 (Fig. 7). However, female craniectomy mice exhibited a peak in necrosis in both the ipsilateral and contralateral cortex at day 1, with a delayed increase in apoptosis in the ipsilateral region. Interestingly, we also observed a significant and trending decrease in the apoptotic regulator, Bax, in male and female mice, respectively. We observed that female mice exhibited a pattern of steady GFAP (Fig. 6) and Bax (Fig. 8) expression with increased pTau (Fig. 6) and cell death (Fig. 7), in contrast to male mice, which showed increasing GFAP, steady cell death, and reduced pTau and Bax. This suggests a potential neuroprotective inflammatory response postcraniectomy. Although the precise mechanism remains to be elucidated, our study provides evidence for the confounding effects of craniectomy and anesthesia in animal models of neurological disorders.

Autophagy has been investigated as a therapeutic target in TBI^17,27,45,46^ and is an established regulator of inflammation.^28–30^ We observed a significant decrease in both SQSTM1 and LC3BII in the ipsilateral cortex from male mice at each time point and a significant decrease at day 4 postcraniectomy in female mice, suggesting autophagy inhibition by craniectomy. Indeed, these results are supported by previous literature assessing the effect of anesthesia on mTOR, a regulator of autophagic activity.^41,47,48^ Feedback between autophagy and immune system maintains the BBB integrity by regulating tight junction proteins.^49–52^ Time course changes demonstrated a trending increase in IgG in the ipsilateral cortex of both male and female mice postcraniectomy (Fig. 9). In male craniectomy mice, claudin-5 was significantly downregulated at each time point in the cortices bilaterally. Female craniectomy mice had a significant reduction in occludin and claudin-1 in both regions, with a significant decrease in claudin-5 in the ipsilateral cortex. Given the greater BBB disruption and lack of astrogliosis in craniectomy females compared to males, our results suggest that neuroinflammation was not the primary driver of BBB disruption postcraniectomy. Matrix metalloproteinase-9, one of the factors in BBB disruption, might be activated by 17β-estradiol and restructure the extracellular matrix due to the protruding brain tissue and midline shift.^53,54^ Although this observation presents an intriguing avenue for future investigation, these data suggest enough evidence that the craniectomy procedure perturbed the BBB, which could significantly impact the reliability of studies employing anesthesia and craniectomy to investigate active targeting strategies.

## Conclusion

Surgical procedures, including anesthesia and craniectomy, contribute to neurological deficits and pathophysiological dysfunction. Our results demonstrated that in the absence of lesion or cavity formation postcraniectomy, cranial defects inadvertently induced structural deficits, including midline shifting. Indeed, craniectomy mice elicited a considerable inflammatory response, which affected neuronal function, cellular homeostasis, and BBB integrity. Although the precise mechanism remains to be elucidated, our study provides evidence for the confounding effects of anesthesia and craniectomy in animal models of neurological disorders. Thus, limiting translational insights for the experimental models of TBI, and they should be combined with naïve mice. Additionally, our study supports the need for more widespread use of closed-head injury models in translational TBI research.

## Data Availability

Data will be made available on request.

## Abbreviations Used

ANOVA: Analysis of variance
BBB: Blood-brain barrier
BSA: Bovine serum albumin
DI: Discrimination index
DAPI: 4',6-diamidino-2-phenylindole
ECL: Enhanced chemiluminescence
FSEMS: Fast spin-echo multislice sequence
GFAP: Glial fibrillary acidic protein
IB: Immunoblotting
IHC: Immunohistochemistry
JNK: c-Jun N-terminal kinase
LC3BII: Microtubule-associated protein 1A/1B light chain 3B-II
Iba1: Ionized calcium-binding adapter molecule 1
IgG: Immunoglobulin G
MAPK: Mitogen-activated protein kinase
MRI: Magnetic resonance imaging
mTOR: Mechanistic target of rapamycin
NOR: Novel object recognition
PBS: Phosphate-buffered saline
pTau: Phosphorylated tau
PVDF: Polyvinylidene fluoride
RT: Room temperature
SBDPs: α-II spectrin breakdown products (SBDPs)
SQSTM1: Sequestosome-1
TBI: Traumatic brain injury
TBS: Tris-buffered saline
TBST: Tris-buffered saline + 1% Tween® 20
