# Supplementary material for: Structural Defects Associated with Craniectomy Induce Neuroinflammation and Blood–Brain Barrier Permeability
Source: Neurotrauma Rep. 2025 Jul 26;6(1):586–99. doi: 10.1177/08977151251362176 (PMC12419447; doi:10.1177/08977151251362176)
Supplement: Supplementary Data [file 08977151251362176_supplementary_data.docx]

**Supplementary Information**

Structural defects associated with craniectomy induce neuroinflammation and blood brain barrier permeability

^a^ Department of Biological Systems Engineering, University of Nebraska — Lincoln

Aria W. Tarudji

Department of Biological Systems Engineering, University of Nebraska — Lincoln

4240 Fair St., Lincoln, NE, 68583

264 Morrsion Center

[Atarudji2@unl.edu](mailto:Atarudji2@unl.edu)

Brandon Z. McDonald

Department of Biological Systems Engineering, University of Nebraska — Lincoln

4240 Fair St., Lincoln, NE, 68583

264 Morrison Center

[Brandon.mcdonald@huskers.unl.edu](mailto:Brandon.mcdonald@huskers.unl.edu)

Connor C. Gee

Department of Biological Systems Engineering, University of Nebraska — Lincoln

4240 Fair St., Lincoln, NE, 68583

264 Morrison Center

[Connor.gee@huskers.unl.edu](mailto:Connor.gee@huskers.unl.edu)

Evan T. Curtis

Department of Biological Systems Engineering, University of Nebraska — Lincoln

4240 Fair St., Lincoln, NE, 68583

264 Morrison Center

[ecurtis@huskers.unl.edu](mailto:ecurtis@huskers.unl.edu)

Forrest M. Kievit* (Corresponding Author*)

Department of Biological Systems Engineering, University of Nebraska — Lincoln

4240 Fair St., Lincoln, NE, 68583

268 Morrison Center

[Fkievit2@unl.edu](mailto:Fkievit2@unl.edu)

**Supplemental Materials and Methods**

**Equation S1. Discrimination index**

$$DI=\left( tNO-tFO \right)\div\left( tNO+tFO \right)$$

Variables:

- DI: discrimination index
- tNO (s): total exploration time for novel objects
- tFO (s): total exploration time for familiar objects

**S1. Cryosection Preparation**

Seven days post-craniectomy, male mice were perfused with 4% PFA, and brains were post-fixed in 4% PFA for 24 hours. Following two 5-minute PBS washes, brains were cryoprotected in 30% sucrose for 72 hours. The brains were coronally sectioned at 2 mm posterior to bregma, embedded in OCT over a methanol-dry ice slurry, sliced into 15 μm sections, and laid on Polysine™ microscope slides (Epredia, Kalamazoo, MI). Slides were stored at -80°C until immunostaining.

**S2. Lysate Preparation**

Mice were perfused at 1, 4, and 7 days post-craniectomy. Ipsilateral and contralateral cortices were homogenized in 300 μL of RIPA buffer (50 mM Tris HCl pH 8.0, 150 mM NaCl, 1% Triton X-100, 0.5% Na Deoxycholate, 0.1% SDS, 1 mM EDTA, 0.5 mM EGTA, 1 mM PMSF, 1 mM Na3VO4, 1 mM NaF) using a TissueLyser II (Qiagen). The samples were horn-sonicated (20s, 20% pulse) and centrifuged (17,740 rcf, 4°C, 5 min). Supernatant protein concentration was determined by BCA assay. Lysates were prepared with β-Mercaptoethanol and 4x Laemmli Buffer (1:9), boiled (95°C, 5 min), and stored at -20°C.

**Supplementary Figures**

Figure S1. Brain images for 1, 4, and 7 days post-craniectomy.

Figure S2. Craniectomy procedure did not induce lesions or cavities.

Figure S3. Craniectomy does not affect exploration during novel object recognition assessment.

Figure S4. Craniectomy procedure induces reactive astrocytes and activated microglia.

Figure S5. Representative brain images at 1, 3, and 7 days post-mild controlled cortical impact (mCCI).

Figure S6. Urinary 8-isoprostane level in naïve and craniectomy mice

Figure S7. Body weights of naïve and craniectomy mice.


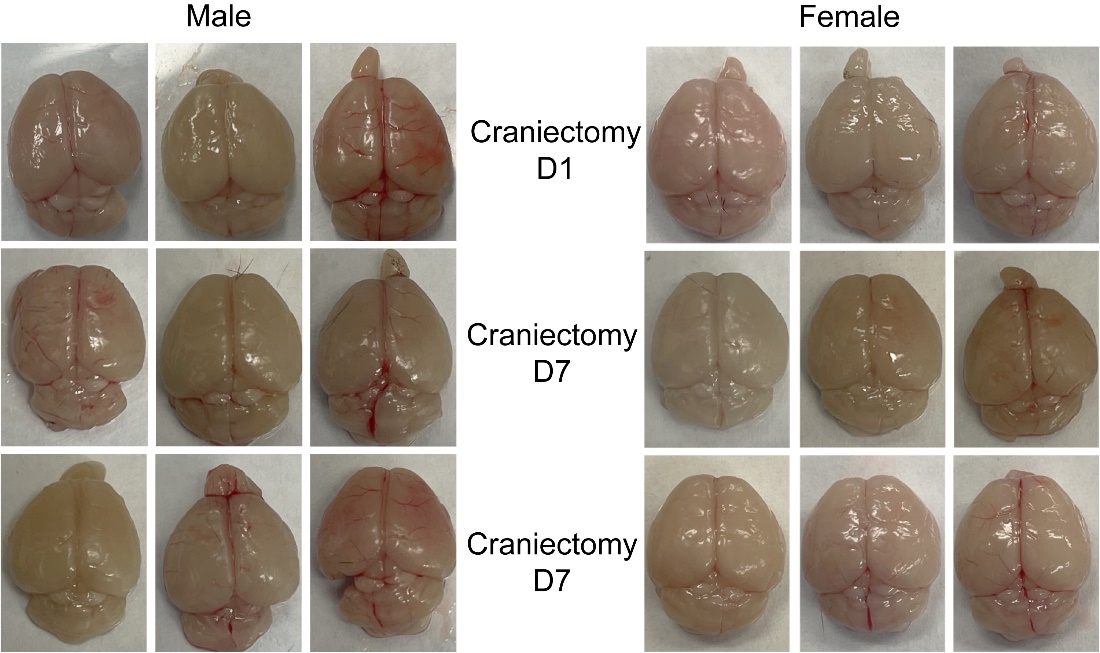


**Supplemental Figure 1: Brain images for 1, 4, and 7 days post-craniectomy.** No visible hemorrhaging or lesion were observed immediately following perfusions for all mice. Brains were collected and used for immunoblotting (IB).


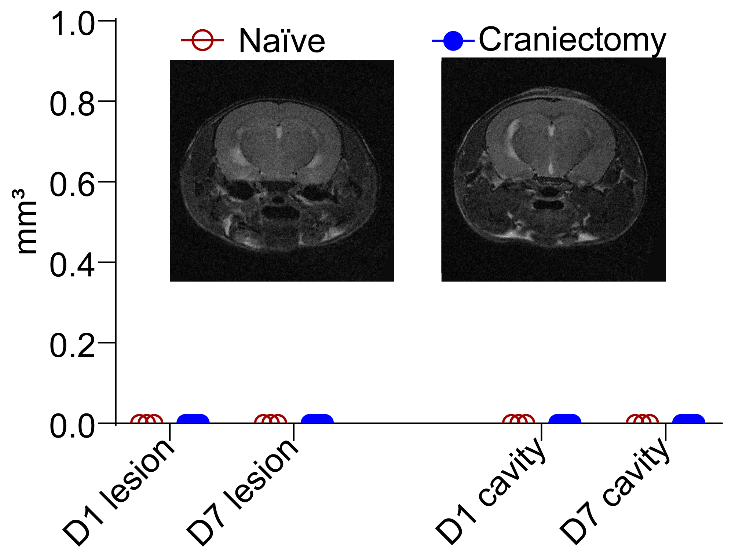


**Supplemental Figure 2.** **Craniectomy procedure did not induce lesions and cavities.** Utilizing T2-weighted imaging, we observed neither lesions nor cavities on day 1 and day 7 post-craniectomy. Data are shown as mean ± SD. One-Way ANOVA statistical analysis was performed.


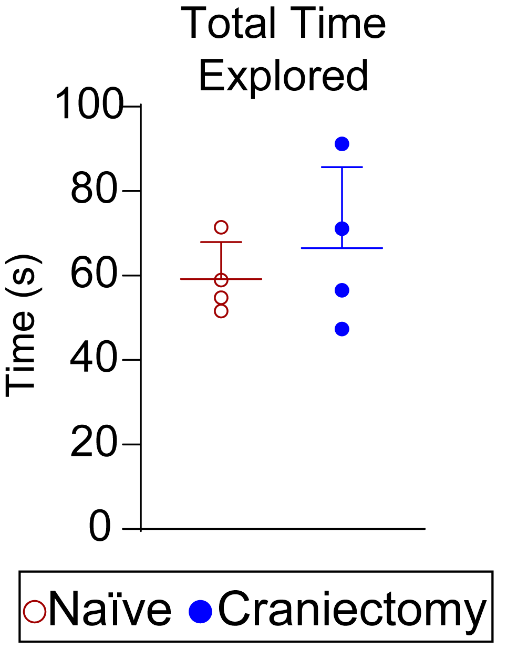


**Supplemental Figure 3.** **Craniectomy does not affect exploration during novel object recognition assessment.** We observed no significant difference in total time explored during object trials, with a trending increase from craniectomy mice. Data are represented as mean ± SD. Student t test with Welch’s correction for post-hoc analysis was performed.

**
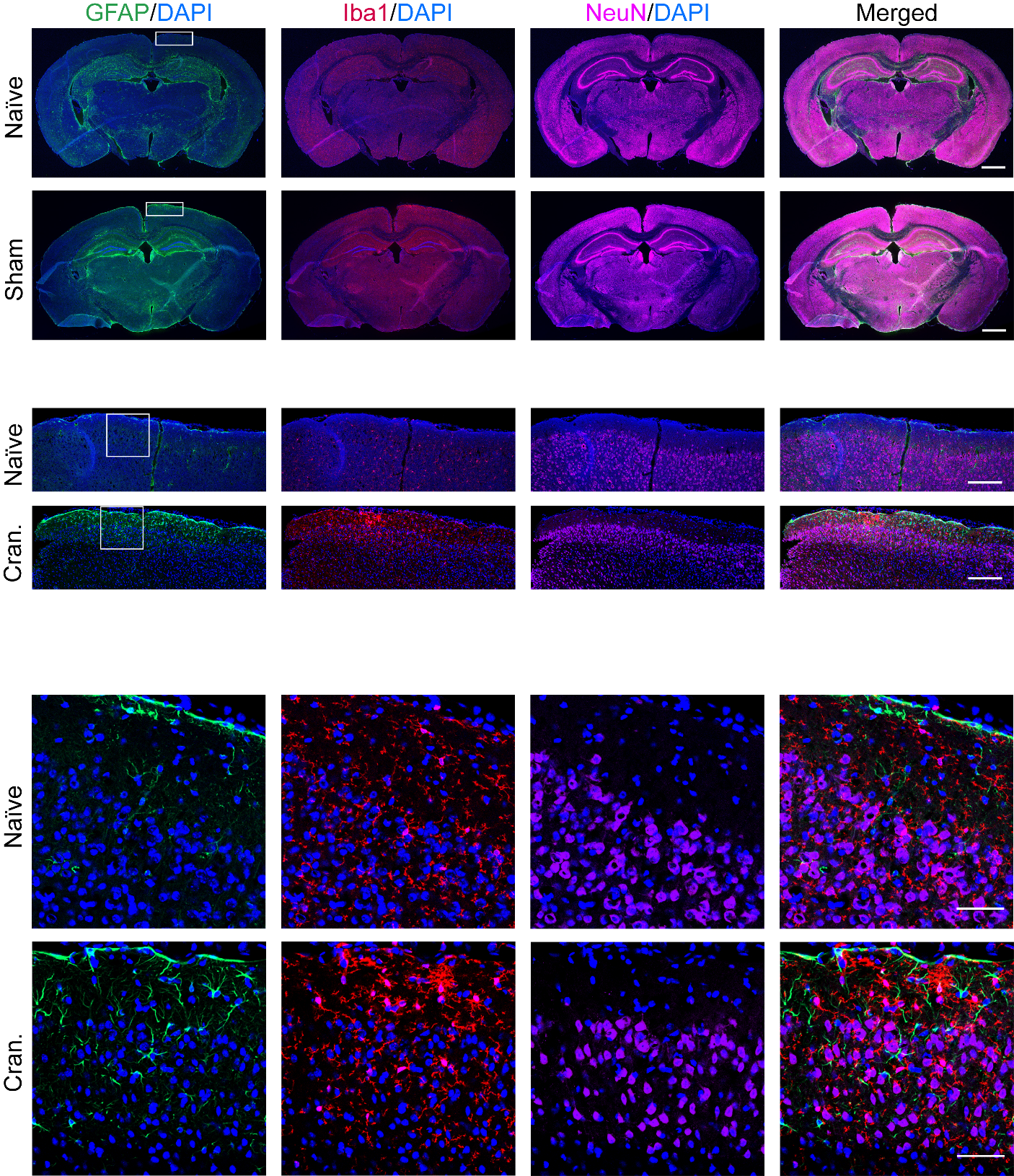
**

**Supplemental Figure 4. Craniectomy procedure induces reactive astrocytes and activated microglia.** Fluorescence microscopy of naïve and day 7 craniectomy mice with GFAP (astrocyte), Iba1 (microglia), NeuN (neuron), and DAPI (nuclei) staining taken with a 5x magnification lens (A), 10x magnification lens (B), and 20x magnification lens (C). The boxed area represents the further magnification area of the cortex. Scale bars: 1000 µm (A), 200 µm (B), and 50 µm (C). Color code: Green-GFAP; Red-Iba1; Magenta-NeuN; Blue-DAPI.


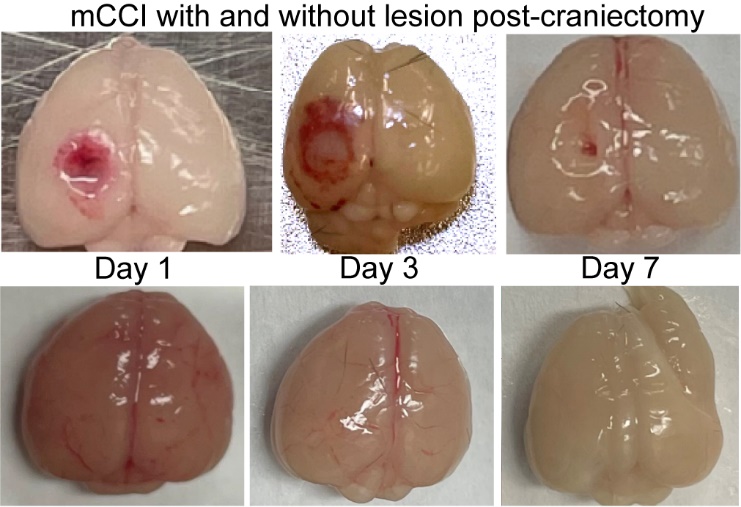


**Supplemental Figure 5. Representative brain images at 1, 3, and 7 days post mild controlled cortical impact (mCCI) (1, 2)**. Brain images following preliminary craniectomy using a 5 mm trephine drill. (Top) Lesion and hemorrhaging were present at 1 and 3 days post-mCCI, with the formation of a cavity at day 7 as a result of the dura disruption during craniectomy. (Bottom) No lesions or hemorrhaging were present following the modified craniectomy procedure that prevents dura disruption.


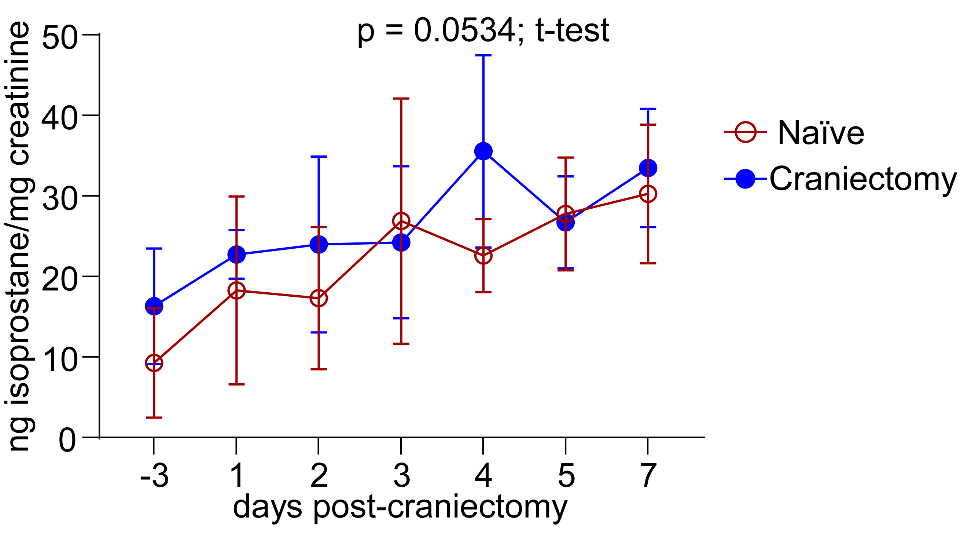


**Supplemental Figure 6. Urinary 8-isoprostane level in naïve and craniectomy mice.** We did not observe a significant difference in the urinary 8-isoprostane levels between naïve and craniectomy mice. However, there is a trending increase in urinary 8-isoprostane levels of craniectomy mice on day 4 post-craniectomy compared to those of the naïve mice. Data are shown as mean ± SD. Student t-test statistical analysis was performed on each corresponding day.

**
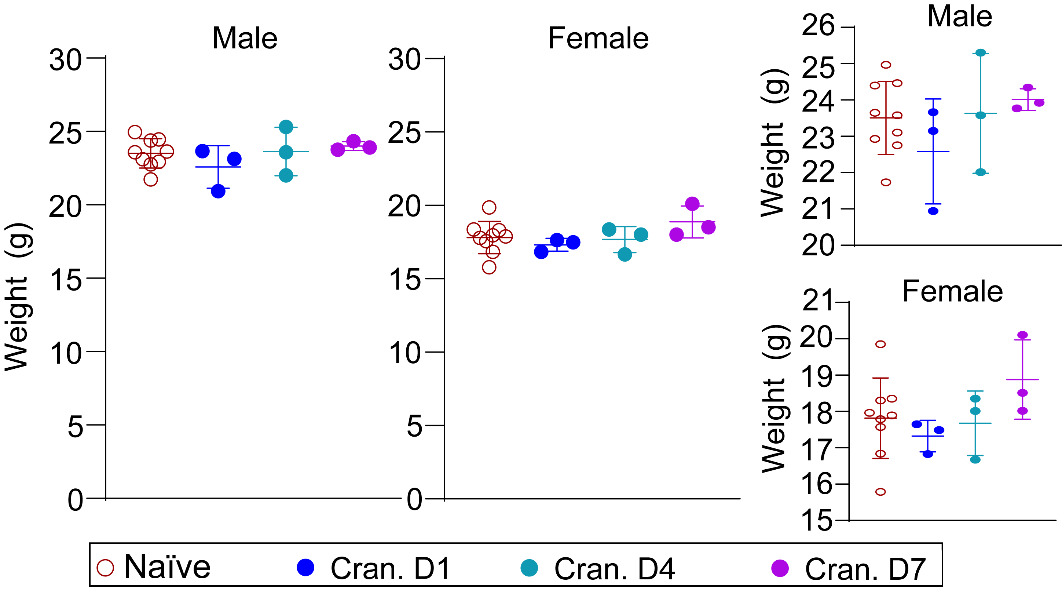
**

**Supplemental Figure 7. Body weights of naïve and craniectomy mice.** No significant differences were observed in body weight for male and female mice post-craniectomy, with a trending increase in bodyweight over time, suggesting craniectomy did not adversely impact general health and well-being. Plots to the right are scaled plots from the left and shown to better identify the individual weights of each animal.

**References**

1. McDonald BZ, Tarudji AW, Zhang H, Ryu S, Eskridge KM, Kievit FM. Traumatic brain injury heterogeneity affects cell death and autophagy. Experimental Brain Research. 2024:1-14.

2. Chen Y, Mao H, Yang KH, Abel T, Meaney DF. A modified controlled cortical impact technique to model mild traumatic brain injury mechanics in mice. Front Neurol. 2014;5:100.
